# Health Status and Barriers to Healthcare Access among “Son-in-Law Westerners”: A Qualitative Case Study in the Northeast of Thailand

**DOI:** 10.3390/ijerph182111017

**Published:** 2021-10-20

**Authors:** Sataporn Julchoo, Nareerut Pudpong, Mathudara Phaiyarom, Pigunkaew Sinam, Anon Khunakorncharatphong, Rapeepong Suphanchaimat

**Affiliations:** 1 International Health Policy Program, Ministry of Public Health, Nonthaburi 11000, Thailand; sataporn@ihpp.thaigov.net (S.J.); mathudara@ihpp.thaigov.net (M.P.); pigunkaew@ihpp.thaigov.net (P.S.); anon@ihpp.thaigov.net (A.K.); rapeepong@ihpp.thaigov.net (R.S.); 2 Educational Service Unit, Sirindron College of Public Health, Chonburi 20000, Thailand; 3 Division of Epidemiology, Department of Disease Control, Nonthburi 11000, Thailand

**Keywords:** expatriates, Westerners, northeast, Thailand, health, healthcare services, healthcare access, barriers

## Abstract

The northeast of Thailand is well-known as a popular destination where many male Westerners marry Thai women and settle down there. However, little is known about their health and well-being. This study aims to explore the Western husbands’ health status and identify barriers hindering their healthcare access. A qualitative case study was conducted from November 2020 to May 2021. In-depth interviews and focus group discussions with 42 key informants who were involved with social and health issues among these expatriates were carried out. The social determinants framework was adapted for guiding the interviews. Data were triangulated with field notes, document reviews, and researchers’ observations. Inductive thematic analysis was applied. Results showed that most male expatriates who married Thai women in the northeast were in their retirement years and had non-communicable diseases, health risk behaviors, and mental health problems. Most of them did not purchase health insurance and held negative impressions toward Thai public hospitals’ quality of care, which was denoted as the main barrier to accessing healthcare services. Other significant barriers consisted of high treatment costs commonly charged by private hospitals and language issues. While the improvement of healthcare quality and the provision of friendly health services are important, public communication with foreign residents, especially male expatriates, is recommended to increase understanding and improve perceptions of the Thai healthcare systems. A regular population-based survey on the health and well-being of expatriates in Thailand, a cost study of a health insurance package, a survey study on willingness to pay for health insurance premiums, and a feasibility survey exploring the opportunity to establish either voluntary or compulsory health insurance among this group should be undertaken.

## 1. Introduction

International migration is a trend that continues to increase every year. The World Migration Report 2020 estimated that the number of people who crossed borders was close to 272 million worldwide, accounting for 3.5% of the global population [1]. The globalized economy creates global flexibility and mobility in workforces, and has consequently led to a significant jump in migration and expatriation [2]. As of 2017, the global expatriate population was defined as an individual who leaves their place of birth to reside in another country with a specific goal, e.g., to work, study, retire, have a new family and reside in another country for a certain duration (normally using a three-month benchmark) [3]. This population amounted to 66.2 million and was estimated to reach 87.5 million by 2021 [2].

The health and well-being of all people around the world is considered fundamental to achieving Universal Health Coverage (UHC). This idea was highlighted by the Sustainable Development Goals (SDGs) with the principle of “leaving no one behind” [4]. Given the substantial amount of migration and number of expatriates, healthcare for this group has become a significant issue. Hence, expatriates’ health is of concern from the perspectives of policy makers and health practitioners. Expatriates have long been considered as one of the vulnerable populations because some have precarious migration statuses, live in substandard conditions, and are unfamiliar with the healthcare system and culture in the host country. International literature has confirmed these findings. For instance, a study among Portuguese expatriates in Angola and Mozambique revealed that one-third of expatriates faced psychological stress [5]. Moreover, 64% of them reported having psychological symptoms, and 20% needed medical assistance [6]. Similarly, a study in Saudi Arabia also revealed that expatriates were likely to have poor mental health [7,8], while another study indicated that road traffic accidents was the top health risk among expatriates in Saudi Arabia [9].Apart from experiencing health problems, expatriates’ difficulties in accessing health services in the host country has also been an issue. These difficulties consist of several barriers such as cultural and language differences and inadequate medical infrastructure [10,11]. For instance, Asian expatriates in the Middle East with underlying chronic illnesses such as diabetes or asthma were reported to face difficulties in accessing health services in the host country as the medical infrastructure was generally tailored to its citizens [12].

Southeast Asia is one of the most economically dynamic regions in the world [13]. As one of the countries in this region, Thailand plays an important role in international migration and has become an expatriation hub due to its geographical location in the middle of the Indo-China peninsula [14]. According to recent data (as of June 2021) from the Ministry of Labour of Thailand (MOL), the number of expatriates with work permits residing in the country amounted to 2,380,767 people, accounting for 3.4% of the total Thai population of about 70 million [15]. The majority of this group (1,618,427 people) were workers from Cambodia, Lao PDR, Myanmar, and Vietnam (CLMV migrants), and accounted for 68.0% of the total expatriates, and 2.3% of the Thai population [15]. Approximately 80,000 expatriates (0.1% of the total Thai population), stayed in the country as retirees, which were mostly English, followed by American, German, Chinese, and Swiss, respectively [16]. It should be noted that the definition of expatriates used in this source may not be the same as used in this present study. In addition, it is widely accepted that it is not possible to have correct information about significant number of expatriates, particularly those who are undocumented and/or do not work in a formal sector [1]. Due to Thailand’s needs for CLMV migrants to help foster its economic development, the Thai Government has introduced policies to protect these people’s health. For example, the Ministry of Public Health (MOPH) implemented a national public insurance scheme in 2013 called the Health Insurance Card Scheme (HICS) for CLMV migrants who work in the informal sector [17]. The HICS covers comprehensive health benefits including inpatient (IP) care, outpatient (OP) care, high-cost treatments, health promotion, and disease prevention activities [18]. However, it appears that the Thai Government has not yet provided a clear direction for protecting the health of expatriates who do not fall under this category as the number of this group of people are relatively small when compared to CLMV migrants. There is a lack of data information about these people, yet data are important to be used for strategic planning in order to improve health and well-being of all non-Thais in the country, as laid out in the vision statement of the National Health Security Office that the health of all people living in the country will be protected [19]. Moreover, several Thai people, including the Government, perceive that these people are wealthy and able to pay for healthcare cost by themselves. Hence, this group of people seemed to be neglected. For instance, certain expatriates may have work permits but do not work in the formal sector and are therefore not covered by the Thai Social Security Scheme (SSS), a public health insurance scheme for workers in the formal sector regardless of nationality [17]. Furthermore, the Thai Government does not have any mandatory policies for retired expatriates to obtain health insurance for their visa renewal (non-immigrant OA or long stay) [20].

In terms of health problems among non-CLMV expatriates in Thailand, the amount of research exploring this group’s health and well-being is still limited. Most studies involving expatriate’s health problems in Thailand revolved around Japanese and Western expatriates. For instance, a study on health service use among Japanese long-stay retirees in Thai tourist provinces revealed that most had underlying chronic diseases, such as hypertension, diabetes, and cardiovascular diseases [21]. Furthermore, a report produced by the Chiang Mai Expats Club and Lanna Care Net found that most expatriates were retirees from Western countries and a large proportion of them did not have any health insurance [22]. It was reported that 95% of them were of old age and had to seek medical care quite often, whereas there was no Government safety net for them and they were living on low pension [22]. Scuzzarello conducted a study in older Westerners in Thailand and found that many retirees could not afford private health insurance due to old age and chronic diseases [23]. The recent study by Khunakorncharatphong et al. (2021) analyzed health service utilization among expatriate patients in Thailand using hospital records of the MOPH during 2014–1018 [24]. It was reported that, for outpatient services, most expatriate patients were female, CLMV at working age, and used the services in the central region [24]. However, when looking at inpatient services, most admitted expatriates were the elderly with noncommunicable diseases (NCDs), and were admitted to hospitals in the northern region more than in other regions [24].

Despite having studies on the health of expatriates in Thailand, most of them were conducted in Bangkok or tourist areas, while research on expatriates in the northeastern region is quite scarce. This region is of particular interest because it is the largest area in the country and is a popular destination for extended stays among Western travelers [25]. More importantly, it is notable for the “Western husband”—mostly, the retired men or businessmen, who marry Thai women and settle down in the region [26,27]; the Western husband is known as “Keui farang” in Thai, which literally means “Caucasian son-in-law”—with the term “Farang” referring to Caucasians. In 2009, a local survey was conducted to estimate the approximate number of son-in-law Westerners, who had families and lived in Udon Thani, one of provinces in the northeast well-known for these matrimonies. It found that there were around 5700 Westerners from 33 different countries spread across 20 districts within the province [28].

However, most of the existing studies about Western husbands in the northeast region focused on societal and cultural factors in relation to transnational marriage rather than health aspects [29,30]. Therefore, the objective of this study is to explore the health status and perceived barriers hindering healthcare access among expatriates—and specifically the son-in-law Westerners—in the northeastern region of Thailand.

## 2. Materials and Methods

**Figure 1:**
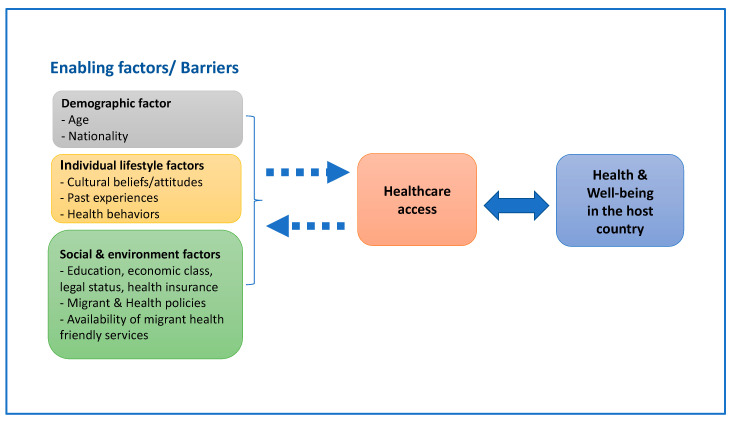
Conceptual framework for guiding the interviews and focus group discussions.

This study adapted the World Health Organization’s (WHO) concept of social determinants of health for framing our interview guide regarding enabling factors and barriers to healthcare access as shown in Figure 1 below. The interviews with Western husbands in the northeast focused on all relevant enabling factors and/or barriers to healthcare access in Thailand—which ultimately influence their health and well-being in the country. These included three groups of factors: demographic factors (such as age and nationality); individual lifestyle factors (such as, cultural beliefs/attitudes, past experiences, and health behaviors); and social and environment factors (such as education, economic status, legal status, health insurance, Thai Government policies in relation to migrant and health, and availability of friendly health services that they could access). The semi-structured interview questions can be seen in the Supplementary File S1.

Besides interviews with Western husbands themselves, we also conducted the interviews with other relevant stakeholders whose work related to the issues around health and other social and environment factors that could affect their living status; this included MOPH policy makers, officers of other Ministries, NGO staff, Academia, and local public health officers in different levels of the health services in both public and private sectors. This helped us have perspectives from different angles, which would be very useful for informing policy design to improve the Thai health system.

For interviews with other key informants (KIs) who were not Western husbands, the researchers placed an emphasis on government policy directions toward expatriate well-being, such as existing legal mechanisms pertaining to their living status, health insurance and availability of supportive services, and views and attitudes of KIs towards the optimal approach that the Thai Government should implement in order to support expatriates’ health and well-being (see interview questions in Supplementary File S1).

### 2.1. Study Design and Setting

This study employed a qualitative case study design. Data were collected via in-depth interviews and focus group discussions (FDGs) conducted among key stakeholders in relation to expatriates’ health and well-being from November 2020–May 2021. All interviewees were purposively selected among relevant stakeholders, who had experiences of working in relation to health and well-being of international migrants and/or foreigners in Thailand as well as male expatriates themselves. Interviewees included policy makers, immigration bureau officers, non-governmental organization (NGO) officers, local healthcare providers (both public and private), and expatriates.

### 2.2. Study Population

The study population consisted of expatriates who married Thai citizens or lived together as partners or had family in the northeast of Thailand. There was a lack of data for this group, particularly health information, and they appeared to be underestimated by the Thai Government due to being smaller in number in comparison to CLMV migrants. The study areas comprised the three provinces, namely Nong Bua Lam Phu, Udon Thani, and Khon Kaen, which were well-known for having many Western husbands.

### 2.3. Data Collection

As mentioned above, in-depth interviews and FDGs were the main methods used for data collection. Purposive sampling was used to identify KIs, and additional informants were also included by snowball selection. Semi-structured questions were employed for the interviews, which took approximately 40–60 min per each interview. The interviews took place in the interviewees’ workplace, at the male expatriate’s house or via video calls or the Zoom meeting application as requested by some participants.

FGDs were held with approximately four informants per group, using the same question as used for in-depth interviews, and two FDGs were conducted: (1) among local primary healthcare providers who had the chance to meet with expatriates and their families in the study area (see Table 1, F5–F8); and (2) committee members who produced the MOPH’s healthcare service provision guidelines for foreigners [31] (see Table 1, A2–A5). Both interviews and discussions were recorded and transcribed upon receiving permission from participants. All interviews and discussions were conducted by SJ, NP, and MP. At the end of each day, research team members summarized the interviews and FGDs and discussed the main content and any inconsistent issues to reach a mutual consensus.

At the end, there were 42 interviewees comprising 6 MOPH policy makers, 4 representatives from other ministries that deal with foreigner-related work (Ministry of Justice, Ministry of Foreign Affairs, Ministry of Social Development and Human Security, and Immigration Bureau of the Royal Thai Police Headquarters), 1 NGO staff, 3 academics, 1 Deputy Director of a Provincial Health Office, 1 Director of a District Health Office, 11 public health officers, 4 registered nurses, 2 staff from the international affairs unit of a private hospital, and 9 expatriates comprising 6 different nationalities (2 French, 2 English, 1 Swedish, 1 American, 1 Australian, 1 Canadian, and 1 Swiss). Details are shown in Table 1.

### 2.4. Data Analysis

Inductive thematic analysis was applied. The interviews were transcribed from audio records and coded by themes. The coding and generated themes were produced by SJ and NP by first reading the audio transcription, taking notes, and highlighting sentences and phrases with the same content to form groups. Subsequently, themes were generated, and several codes were introduced as subsets of the themes. Interview data were triangulated with field notes, document reviews, and researchers’ observations.

Ethical approval to conduct the study was obtained from the Institute for Human Research Protection, Thailand (IHRP 036/2563). The data collection process strictly followed the Declaration of Helsinki. The informed consent process was approved by the IHRP. All informants were provided with a participant information sheet and informed consent was granted prior to conducting the interview and survey.

## 3. Results

Three themes were identified from the interviews and FGDs as key determinants in hampering access to healthcare services, namely: (1) the health status of Western husbands in the northeast of Thailand; (2) negative impressions toward the Thai healthcare system; and (3) language and financial barriers. Detailed information and transcribed portions from the interviews are presented below.

**Theme 1.**  *Health status of Western husbands in the northeast of Thailand.*

### 3.1. Non-Communicable Diseases (NCDs), Health Risk Behaviours, and Mental Health Were Issues of Concern

Most of the expatriates were retirees and held retirement visas for living in Thailand.

“*First, I came here for holiday then I met my girlfriend. Then we got married and moved to Australia for a lot of years. Then, when I retired, we decided to settle down here*.”

(I1)

Several participants also reported that most male expatriates had chronic diseases such as hypertension, diabetes mellitus, and cardiovascular diseases. This can be seen via sample statements below.

“*Male or female Westerners who have family in Thailand* *mostly have chronic diseases because they are elderly people*.”

(A2)

“*I have a heart infarction, blood pressure, sugar in blood*”

(I3)

Moreover, some expatriates reported having health risk behaviors such as smoking and drinking.

“*I saw that some of them drank beer and smoking day by day. It’s their lifestyle. They were retired and didn’t work nowadays. They can do everything whatever they want, even though they have emphysema. They should quit smoking but they don’t. Whenever their conditions are out of control, it makes them sick*.”

(G4)

Apart from physical health problems, expatriates also had mental health problems. Some expatriates seemed to be unable to adapt to Thai culture and society. Coupled with isolation from a familiar way of life and societal norms, this may have played a role towards their mental health issues. It was also reported that some expatriates had alcoholism and depression, eventually leading to suicide.

“*When they come to Thailand, they come with some dreams. But it might not turn out as they hoped for. Some of them broke up, got dump, and finally ended relationships with their girlfriends. It’s a private life problem of them*.”

(D1)

“*They drink alcohol and get drunk. This complicated situation had also resulted in family violence, mental health problem, leading to suicide*.”

(E1)

### 3.2. They Preferred to Pay Out-of-Pocket Rather Than Buying Health Insurance

All expatriates noted that they were comfortable paying out-of-pocket when requiring health services instead of buying health insurance. Some of the reasons for not buying health insurance are as follows.

First, even though most of them were the elderly, living with NCDs, risky health behaviors, and mental health issues, they did not have a long-term plan for their health as they perceived that they did not need any healthcare services and thus ignored purchasing health insurance.

“*I’m still healthy. I don’t have to take medicine regularly*.”

(I2)

“*I hadn’t gotten any health insurance here so far with my retirement visa. I’m not required to have a health insurance*.”

(I1)

Second, expensive health insurance premiums were crucial in their decision to not buy health insurance. As most expatriates were in their retirement years (more than 55 years old), the cost of insurance was higher than other age groups and become more expensive the more the person aged.

“*Most of them are the elderly, the premium for health insurance is approximately 3033 USD per year. They don’t have money to pay for health insurance*.”

(F5)

“*It’s hard to get health insurance when you’re 72 years old. It’ll be too expensive for me to get it so I put my money in my bank account for health*.”

(I9)

Third, although some male expatriates had health insurance from their home countries, it did not cover health services provided in Thailand. Some expatriates stated that when they were seriously ill, they would rather return to their home countries instead of finding medical care here.

“*But I also ever thought if I got sick one day, I just jump to the airplane and go back home, because I have health insurance there. That’s easy. It’s easier than doing it here*”

(I1)

**Theme 2.**  *Negative impressions toward the Thai healthcare system.*

*Several negative perceptions among expatriates dissuaded them from seeking healthcare services from Thai healthcare providers.*

### 3.3. Large Crowds and long Waiting Times in Thai Hospitals

Healthcare providers and expatriates both commented that most expatriates had negative views toward Thai public hospitals due to the large crowds and long waiting times.

“*I had to be at the hospital at 5 o’ clock, which means I had to get up since 4 o’ clock. Around 6 or 7 o’ clock I went home for my daily routine and I got to see the doctor at the 11 o’ clock. I may be sick and sicker. It took so much time (laughing). That’s why I go to the clinic. It’s been a long time since I went to the hospital*.”

(I3)

### 3.4. Lack of Hygiene in Thai Hospitals Relative to Their Home Countries

Some key informants said that Thai public hospitals seemed less hygienic because of the large crowds and relatively old facilities.

“*Some foreigners don’t use health services in public hospitals because the Thai hospital environment doesn’t have good hygiene; old buildings, crowded patients. In their attitude, the hospital doesn’t clean, they could sick when using health services in this place*.”

(H1)

### 3.5. Mistrust of Medicine and Service Quality

First, some expatriates were concerned that the medicines provided by Thai hospitals may be of lower quality than the equivalents prescribed in their home country. In the event that they had to visit a doctor other than their personal doctor, they chose to take brand-name drugs from their home countries rather than generic medicines.

“*They don’t ask about the prescription of blood pressure drugs that we have... ... They don’t used Thai medicines because they thought that their medicines in their homeland are superior to Thai medicines.*”

(H1)

In some cases, expatriates chose to buy medicines from a pharmaceutical store instead of visiting a hospital because they could purchase the same medicines as in their home countries.

“*Some of them asked me for medicines for chronic diseases (which were not available at Thai district health centers). So, they choose to buy drug from pharmacies instead. The Westerners in this area intend to use only original medicines*.”

(F5)

Second, some of them raised concerns about the quality of health services at Thai providers. For example, an expatriate who had received treatment in a hospital mentioned that his wife’s symptoms did not improve until he decided to visit a private hospital and received treatment from another doctor.

“*Here is an example. My wife had a Thyroid gland come up here, so we’ve been to the doctor in Hospital X, they gave a medicine for the first time, came back one month just to check the blood, came back one month to do something else. It took three months. So later we took her to Hospital Y, the doctor said “Why didn’t you come here straight away? Those doctors are stupid.” Bang…she (doctor) took the fluid away and it’s gone. We have to go to see the doctor again this month, but it’s already fixed*.”

(I1)

**Theme 3.**  *Language and financial barriers were key determinants hampering access to care.*

### 3.6. High Cost of Treatment Charged by Private Hospitals

Some key informants identified that male expatriates preferred visiting private hospitals or clinics rather than public hospitals because of supposedly higher quality, faster, and more convenient healthcare services.

“*The healthcare service cost in Thailand less expensive in foreign country. The private hospital is the best way to obtain the healthcare service in Thailand because of affordable price, feel more at ease, and easy access*.”

(E2)

However, it appeared that expatriates suffered from expensive health services in private hospitals in Thailand even though they were supposedly cheaper in Thailand.

“*The private hospitals in Thailand like Farangs because they can charge 10 times more than they charge the Thai person. I was there some years ago because I had an accident. They charged me 30,000 Baht (905 USD) per day. What they did just to put fluid inside. You’d heard a lot of stories from Farangs here that they got stuck to get out of the hospital. They want millions of Baht. It’s really crazy*.”

(I1)

### 3.7. Language Was a Significant Barrier for Both Healthcare Providers and Expatriates

A major obstacle to healthcare access was language. Communication is vital to diagnosing any illnesses or disorders accurately and promptly. However, experiences from the sample group of expatriates showed that many were unable to communicate in English and did not understand Thai. On the other hand, many Thai healthcare personnel were not competent in any language other than Thai. Therefore, even if the Thai staff were proficient in English, it would not have mattered as their patients’ native tongues were in other languages.

“*The nurse didn’t understand English, but in the end, you found someone who understands English and tries to help you, but they cannot hold a conversation just hello and hi instead of phrases…. Some nurses they know my symptoms and my conditions in Thai, but they cannot express in English*.

(I9)

“*Expatriates from Germany, they don’t speak English but they speak only Deutsch*.

(F5)

Most of the time, expatriates’ wives acted as translators between the healthcare staff and patient at the hospital.

“*I got hands so I could use body language…. Besides that, my wife helps me*.”

(I1)

Some interviewees added that although the attending healthcare professional could not speak English, sometimes there were other staff who could help translate.

“*There’s always somebody there who can speak good enough in English. That’s enough to get by. No problems*”

(I1)

**Figure 2:**
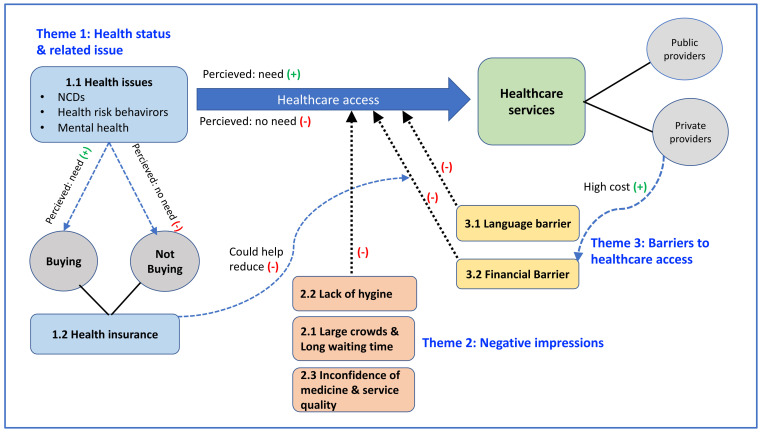
Summary of the study results.

The summary of the study results is shown in Figure 2. The male expatriates or the son-in-law Westerners in living in the northeast of Thailand were found to have NCDs, health risk behaviors, and mental health problems. However, to seek health care or access to care or not may depend on their perceived health needs. This could be hampered by language barrier and financial barrier. The perception of high hospital fees charged by private hospitals would increase the financial barrier. In addition, negative impressions of the Thai health system, namely lack of hygiene, large crowds and long waiting times, could also worsen their desire access to health services. Most of them did not have health insurance, and the reason for purchasing it or not depended on their perceived need. However, having health insurance could help reduce their financial barriers in accessing health services.

## 4. Discussion

Overall, the study found that most Western husbands residing in the northeast of Thailand were generally in their retirement age and had chronic diseases. Furthermore, most perceived that they were still in a good shape and rarely visited Thai public hospitals. However, the reasons for not visiting Thai hospitals were not simply because they did not fall ill but it was due to certain negative impressions toward the Thai healthcare system as well. Furthermore, key determinants that deterred healthcare access among these expatriates included expensive treatment costs and language barriers.

A notable observation was that although this study focused on male expatriates who married Thai women, most of this group comprised Western retirees. This finding concurs with several previous studies indicating that Thailand is a popular destination for the retirement of many foreigners around the world [29,30,32]. Furthermore, having a family appeared to be an important reason for settling down in a new country. For example, Miyashita et al. reported that 25% of Japanese chose to retire in Thailand and marry Thai women [21]. Nakai et al. also found that 25.3% of male Japanese expatriates residing in the Philippines and Thailand generally lived with a non-Japanese partner [33]. A study on the health and social welfare of expatriates in Southeast Asia conducted by Wilde and Gollogly also revealed that over 90% of expatriates in Thailand were male and of old age, and some of them married their local partners and raised families there [34]. It is important to note that the male expatriates in this study comprised a mix of working-age expatriates and retired expatriates. The study also found that chronic diseases was the main health problem among the expatriates given their age profile. This finding was in line with the recent quantitative study by Khunakorncharatphong et al. (2021), which analyzed the health service records of the MOPH from 2014–2018 [24]. This study found that most expatriate patients, who were admitted to public hospitals in Thailand, were elderly and had noncommunicable diseases (NCDs); this was found more prevalent in the northern region than in other regions [24]. Diseases most frequently mentioned were hypertension, diabetes mellitus, and cardiovascular diseases. This is to be expected as chronic diseases, and particularly NCDs, are commonly found among older age groups [35]. This finding was consistent with a study conducted by Miyashita et al. which found that 33% of Japanese retirees in Thailand suffered from chronic diseases or sequela, even though that study was conducted in other parts of the country, including Bangkok (central), Chiang Mai (north) Chiang Rai (north), and Phuket (south) provinces [21].

However, other studies have also shown that NCDs among expatriates may occur in more than just retirees. A study in the United Arab Emirates (UAE) indicated a high prevalence of obesity and associated NCDs among working expatriates, suggesting that they may have led unhealthy lifestyles [35]. Another study of Indian expatriates living in Saudi Arabia reported a high prevalence of smoking in those aged 41–50 years (42%) and over 51 years (7%) [36]. Therefore, the same explanation may also apply to our study because some expatriates in the northeast of Thailand were heavily involved in health risk behaviors such as smoking and drinking. Hence, this information may help inform local healthcare providers in Thailand on how to play an active role in health promotion and prevention of NCDs among expatriates to help reduce NCD burden [37].

Most of the expatriates in this study preferred to pay out-of-pocket rather than purchasing or using health insurance when accessing health services. This is similar to previous studies among retired expatriates in other parts of Thailand, which also found that expatriates did not usually have health insurance [22,23]. Another study conducted among Korean expatriates living in Vietnam, Cambodia, and Uzbekistan by Kim et al. corroborates this result as well; it showed that only approximately 22% of Korean expatriates possessed health insurance [38]. Furthermore, a study in Saudi Arabia by Alkhamis et al. found that 30% of expatriate employees were uninsured or had not yet enrolled in any health insurance schemes, and 79.4% of these uninsured expatriates did not have valid reasons for remaining uninsured [39].

The lack of health insurance for expatriates is also a concern since most of them are considered elderly. If they were to fall severely ill, their families may experience catastrophic health expenditure, subsequently affecting their livelihoods. However, the reasons given for not buying health insurance in this study corresponded with a study conducted by Wilde and Gollogly; it reported that the purchase of health insurance becomes virtually impossible after the age of 70 since health insurance premiums jump substantially with advancing age [34]. Furthermore, it is feasible that substantial healthcare expenses for these households may not arise as some expatriates are still covered under their home country’s health insurance schemes. Thus, if they were to fall severely ill, they would just return home to receive treatment. This phenomenon was also found by Kohno A, et al., and Miyashita et al., which reported that Japanese expat retirees who live in Thailand generally returned to Japan for the treatment of chronic or serious diseases [21,40,41]. Nevertheless, it should be noted that expatriates in the northeast were relative old and might not be able to travel back home right away when they become sick. Hence, the enactment of a compulsory health insurance in Thailand may be a better policy option on a long-term basis, particularly for those who did not have any health insurance and those that the health insurance they bought from the home country could not be applied in Thailand.

Male expatriates in this study perceived that most Westerners were charged extremely high treatment costs by Thai hospitals. This corresponded with a study performed by NaRanong et al., which was a 2003–2008 survey on the price of certain procedures at four private hospitals that provided services to foreign patients. The survey found that the prices of caesarean sections, appendicitis operations, hernia operations, gall bladder operations, and knee joint replacement operations were more expensive than public hospitals and continue [42]. Although the magnitude of the problems of access to care in association with this financial barrier cannot be known from this study, the information should be useful to help inform the Government and Thai healthcare providers that not all Westerners, and by extension expatriates, are wealthy and/or able to pay for high-cost care. Additionally, it might also be an opportune time for Thailand to convince expatriates about the importance of having health insurance to protect themselves and their families from facing potentially dire financial difficulties due to healthcare expenses. The Thai Government and relevant authorities that oversee the health insurance scheme should start thinking about promoting knowledge about health insurance among expatriates as well as designing a comprehensive and holistic public health insurance scheme to protect all residents within the country. As mentioned earlier, the only public insurance scheme for non-Thais focuses on CLMV migrant workers. Therefore, a comprehensive scheme would help address the lack of health insurance for expatriates who have families in Thailand but are not classified as workers.

Negative attitudes toward the Thai healthcare system seemed to permeate throughout the expatriate community in the northeast region. The largest factors contributing to these perceptions included mistrust of the quality of medicines and hospital services, and the presence of non-hygienic environments in Thai public hospitals. This finding was consistent with a study among Japanese retirees in other parts of Thailand, which reported concerns about the quality of medical staff such as doctors and nurses, and other equipment operators and cleaning staff in Thai hospitals [40]. However, this is likely to be a lack of confidence among these expatriates about service quality, even though public hospitals in Thailand have been accredited by the Healthcare Accreditation Institute (Public Organization) or HAI since 1999 [43]. In addition to the HIA accreditation, several public hospitals have also been accredited by an international accreditation institute such as the Joint Commission Institute (JCI). For example, the Queen Sirikit Heart Center of the Northeast in Khon Kaen province and Thabo Crown Prince Hospital in Nong Kai province, both public hospitals, have already been awarded the gold standard by the JCI [44]. Their concerns about the quality of medicines may also be misplaced as the use of non-generic drugs does not necessarily mean low drug quality. In fact, all drugs used in Thai public hospitals must be approved by the National List of Essential Medicine (NLEM) committees [45]. However, while Thai people may feel confidence about the accreditation and approval of Thai hospital service quality, many Westerners may not be aware of this information and even if they know, they still might not feel confident in local standards.

Moreover, at the present, the Thai Government has established a development plan for health sector in order to make Thailand become the global “Medical Hub” [46], which would lead to significant improvement of Thai public hospitals. However, evidence has suggested that private hospitals in Thailand are the main choice of healthcare access among foreign tourists as well as expatriates. For instance, in preparation for being the Medical Hub, the MOPH survey in 2019 found that about 92.7% of foreign tourists visited private hospitals [47]. Similarly, the study among Japanese expatriates by Miyashita et al. also revealed that 87.7% of them used private hospitals or clinics, instead of public hospitals [21]. However, it is important to note that private hospitals or clinics are usually located in the big cities or urban areas. Thus, it may not be so convenient for long-term residents in local villages in some rural areas where most son-in-law Westerners have been living, compared to public hospitals, which are commonly established in every district and subdistrict in Thailand.

The finding of language barriers hampering healthcare access among expatriates was consistent with several previous studies that indicated that language differences were strong barriers to communication between healthcare providers and expatriate visitors in healthcare facilities [34,38,40,41,48,49]. For example, Kohno et al. reported that the language barrier was the most serious issue, affecting healthcare utilization among Japanese retirees living in Malaysia [41]. This suggests that friendly health services should be properly designed and implemented in Thai public hospitals to accommodate expatriates’ health needs. For instance, hospitals with a significant number of foreign patients should provide language services in the form of translators or interpreters. This type of service has already been provided in some premium private hospitals in Bangkok such as providing translators or hiring Japanese-speaking doctor to cater to Japanese patients [21], but has not yet been fully implemented in public hospitals. Additionally, public health providers also hire migrant health workers (MHW) who serve as interpreters in public facilities and migrant communities in regions with a dense CLMV community, such as Samut Sakhon, the densely CLMV migrant-populated province in central region [50]. However, this has not yet been operationalized in other parts of the country, including the northeast.

Although this study was conducted in a small group of expatriates under a specific context in the northeast of Thailand, it has provided additional information that could be useful for several developing countries in moving forward to achieve the UHC and SGDs in the concept of “leaving no one behind”. Evidence from the study showed that even though expatriates are from advanced countries, and are usually deemed to be a wealthy group of people, some still face difficulties while living in unfamiliar environments in less affluent countries, particularly with their health. This issue does need policy attention. Several previous studies on expatriates in developing countries suggest the same direction; for example, U.S. retirees living in Mexico and Panama reported having difficulties in relation to healthcare choices, insurance availability, and quality of care [51]. Most expatriates (73.8%) from North America or Europe living in Western Ghana were found to have health problems, such as diarrhea and acute respiratory infections [52]. Portuguese expatriates in Sub-Saharan Africa had experienced both physical and mental health problems, which required medical attention [5,6]. Thus, in order to reduce barriers for the son-in-law Westerners in accessing the Thai public health services, an increase in public communication to gain confidence in service quality as well as the provision of friendly health services are crucial. Though there is no compelling evidence in this study that physical inaccessibility to healthcare existed amongst the son-in-law Westerners, the evidence about the doubt of service quality in public healthcare, the unfamiliarity with the Thai language and culture, and high out-of-pocket payment (especially when attending private facilities) might lead to “unmet need” for health services. Such a situation causes a problem not only for the son-in-law Westerners alone, but also for the Thai healthcare system, as the system may risk missing people with health need from the care.

The study faced some limitations. Firstly, the study focused on expatriates living in the northeast of Thailand only. Thus, the nature of purposive sampling and the use of certain provinces as study sites may limit generalizability. However, this study did not aim for generalizability in the first place, but rather transferability through the use of a case study approach [53]. Findings from this study would be useful and transferable to other different places to some extent. Secondly, the researchers collected data during the COVID-19 pandemic. Therefore, data collected in this study may not reflect an accurate picture of some other expatriates who travel more frequently due to travel restrictions implemented during this period. This may have resulted in certain biases that affected the outcomes of the interviews. Thirdly, as the son-in-law Westerners are not always physically presenting in the field and we conducted the data collection during the COVID-19 pandemic, which made it difficult to contact them and/or travel to their places, the interview samples were relatively small. However, having triangulated the data with other sources of information, we believe that the use of pragmatic considerations for determining the samples provided good enough data for the study [54]. Fourthly, the study may have also encountered social disability biases since the researchers had to disclose their status as public health personnel or MOPH officers. Hence, it is possible that the interviewees might have tailored their answers in a more favorable light to meet the needs of the researchers.

Hence, to improve the Thai health system and provide high-quality services for international migrants and/or expatriates, we recommend that: (1) the Thai Government should implement compulsory health insurance for all foreigners living in the country to prevent any potential catastrophic health expenditures from afflicting households while also contributing to the achievement of UHC and the SDGs; (2) authorities who work in the field of public health should properly design and/or improve health services that correspond to expatriates’ health needs such as the provision of friendly health services that reduce language barriers and improving language proficiency for public health personnel; and (3) local public health providers, particularly at the primary health care level, should play a proactive role in health promotion and prevention among male expatriates in order to reduce their health risk behaviors, NCDs, and mental health problems. Additionally, in order to improve the health system, there should be future studies: (1) an annual population-based survey to monitor the health statuses of male expatriates in order to have more accurate and updated data; (2) a cost study of a health insurance package for this group; (3) a survey study on willingness to pay for health insurance premiums; and (4) a feasibility study to explore voluntary or compulsory health insurance and the feasibility of its implementation. Though we did not intend to highlight that a provision of insurance was the ultimate tool to address the problem regarding high out-of-pocket payment amongst them, a better comprehension on the insurance alternative for son-in-law Westerners is imperative. Moreover, information retrieved from the annual survey would be helpful for monitoring their health in the long-run, and the results of the cost study, the survey of willingness to pay, and the feasibility study would be useful for policy decisions in designing and implementing a comprehensive and holistic policy to protect the health of expatriates.

## 5. Conclusions

The results showed that most of the Western husbands in the northeast of Thailand were in their retirement age, and had NCDs, health risk behaviors, and mental health problems. Most of them did not purchase health insurance and were not proactive in planning for their long-term health as they believed that they were did not need any healthcare services. Furthermore, many of them had certain negative impressions toward the quality of care at Thai public hospitals and this was a major barrier to accessing health care services. Other barriers to accessing healthcare included the high cost of treatment commonly charged by private hospitals and language issues. While the improvement of the quality of healthcare as well as the provision of friendly health services are of utmost importance, public communication with international migrants and especially male expatriates is highly recommended to increase understanding and positive impression towards the Thai health care system. A regular population-based survey on the health and well-being of expatriates in Thailand, a cost study of a health insurance package for this group, a survey study on willingness to pay for health insurance premiums, and a feasibility survey to explore the opportunity of establishing, either voluntary or compulsory, health insurance among this group of people should also be conducted.

## Figures and Tables

**Table 1:** List of interviewees about health status and barriers to foreigners’ healthcare access.

Code	Involvement with Social and Health Issues among Expatriates
**MOPH Policy Makers**	
A1	Head of Health Security Development, Health Economics and Health Security Division
A2 *	Committee member who took part in the production of the guidelines for healthcare service provision for foreign tourists
A3 *	Committee member who took part in the production of the guidelines for healthcare service provision for foreign tourists
A4 *	Committee member who took part in the production of the guidelines for healthcare service provision for foreign tourists
A5 *	Public Health Technical Officer of the Specific Health Service Development Unit, Health Administration Division, MOPH
A6	Director of a community hospital located in the northeast that provides health services for oversea visitors
**Other Ministry Participants**	
B1	Consultant of the Ministry of Justice
B2	Diplomatic Service Officer of the Consular Affairs Department, Ministry of Foreign Affairs
B3	Inspector of the Udon Thani Immigration Bureau
B4	Officer of the Women and Family Development Leaning Center, Ministry of Social Development and Human Security
**NGO**	
C1	Staff who has experience in work related to health services and quality of life of migrants and/or foreigners
**Academia**	
D1	Researcher with experience in conducting research on gender approach to migration and transnational studies
D2	Researcher with experience in conducting research on health status and quality of life among expatriates in Thailand.
D3	Researcher with experience in conducting research on transnational anthropology with gender sensitivity
**Primary Care Public Health Providers**	
E1	Deputy Director of a Provincial Health Office located in the northeast
E2	Director of a District Health Office located in the northeast
F1	Public health officer of a subdistrict health center in Nong Bua Lam Phu province
F2	Public health officer of a subdistrict health center in Nong Bua Lam Phu province
F3	Registered nurse of a subdistrict health center in Nong Bua Lam Phu province
F4	Public health officer of a subdistrict health center in in Nong Bua Lam Phu province
F5 *	Director of a subdistrict health center in Udon Thani province
F6 *	Director of a subdistrict health center in Udon Thani province
F7 *	Director of a subdistrict health center in Udon Thani province
F8 *	Director of a subdistrict health center Udon Thani province
F9	Director of a subdistrict health center in Khon Kaen
F10	Public health officer of a subdistrict health center in Khon Kaen province
F11	Director of a subdistrict health center in Khon Kaen province
**Community and Provincial Hospital Healthcare Providers**	
G1	Registered nurse of the Healthcare Service Department, MOPH, who is responsible for providing services for foreigners in local public hospitals
G2	Registered nurse of the Healthcare Service Department, MOPH, who is responsible for providing services for foreigners in local public hospitals
G3	Registered nurse at a local public hospital
G4	Registered nurse at a local public hospital
**Private Hospital Staff**	
H1	Staff working in the international affairs unit of a private hospital in Udon Thani province
H2	Staff working in the international affairs unit of a private hospital in Udon Thani province
**Male Expatriates**	
I1	Male expatriate living in Nong Bua Lam Phu province
I2	Male expatriate living in Nong Bua Lam Phu province
I3	Male expatriate living in Nong Bua Lam Phu province
I4	Male expatriate living in Nong Bua Lam Phu province
I5	Male expatriate living in Udon Thani province
I6	Male expatriate living in Udon Thani province
I7	Male expatriate living in Udon Thani province
I8	Male expatriate living in Khon Khan province
I9	Male expatriate living in Khon Khan province

* People who were interviewed in groups.

## Data Availability

Ethical restrictions are imposed by the Institute for the Development of Human Research Protections, Thailand. The provision of complete interview transcripts is prohibited as these transcripts contain potentially identifiable and sensitive information of the participants.

## Supplementary Materials

The following are available online at https://www.mdpi.com/article/10.3390/ijerph182111017/s1, Supplementary File S1.
