# Supplementary material for: Health Status and Barriers to Healthcare Access among “Son-in-Law Westerners”: A Qualitative Case Study in the Northeast of Thailand
Source: Int J Environ Res Public Health. 2021 Oct 20;18(21):11017. doi: 10.3390/ijerph182111017 (PMC8583070; doi:10.3390/ijerph182111017)
Supplement: Supplementary file 1 [file ijerph-18-11017-s001.zip › ijerph-1391789-supplementary.pdf]

Supplementary 1.

I. Guideline questions for interviewing relevant stakeholders regarding taking care of health and quality of life among foreigners who have a family and living in Thailand

- What is the overall situation among foreigners who have a family and living in Thailand? Are there any specific health issues that are different from other migrant groups?

- What is the role of your organization in relation to health and quality of life of foreigners who have a family and living in Thailand?

- What are the pros and cons of having foreigners who came to have a family and living in Thailand? And why?

- In your opinion, would Thai health systems have financial burden due to this situation -- foreigners have a family and living in Thailand? Are there similarities or differences when compared with another migrant group (such as Cambodia, Laos, Myanmar, and Vietnam; CLMV), and how?

- Should the Thai government implement a specific health policy for this group of people?
    - Health insurance e.g., should the Thai government make health insurance available for this group of people? If so, whether the insurance system should be separated or included with existing insurance system (for example, Health insurance card scheme for migrants, which is managed by Ministry of Public Health). In terms of financial management and benefit package, how should it be?
        - Provision of health promotion and prevention services e.g., basic vaccination or non-communicable diseases (NCDs) screening
        - Treatment for communicable diseases or public health threats e.g., tuberculosis or elephantiasis
        - Treatment for NCDs e.g., diabetes and hypertension
    - According to the previous question, who should have the authority for each issue? Is there a legal framework to operate? And who should be responsible for operational expenditure?

- How should the Thai government set up the policies to promote the quality of life of foreigners who came to have a family and living in Thailand e.g., work permits, visa extensions, housing purchases,...?

II. Guideline questions for interviewing male expatriates, who came to have a family and living in Thailand regarding their health and well-being

- What was the main reason that made you decide to live in Thailand? And in your opinion, what are the pros and cons of living here?

- In terms of health, do you or your family members need to receive any health services from health facilities or not? If yes, you mostly go to public facilities or private facilities? Why?

- If you have experiences in receiving health services from any health facilities in Thailand, what do you think about health service provision for foreigners at that health facilities? Please share your experiences.

- Do you think having health insurance is essential for living aboard? Currently, do you and your family members have any health insurance?

- During COVID-19 pandemic, have you been affected by this pandemic? If yes, which aspects have you been affected, and how? Have your plans to travel and/or stay in Thailand been changed because of the pandemic, and how?

- In your opinion, should the Thai government provide care for foreigners residing in Thailand, and how? (Whether in a normal situation and pandemic situation)

Health

- Health insurance e.g., should the Thai government make health insurance available for this group of people? If so, the insurance system should be whether separated or included with existing insurance system (for example, Health insurance card scheme for migrants which is managed by Ministry of Public Health). In terms of financial management and benefit package, how it should be?
  - Health promotion and prevention provision e.g., basic vaccination or non-communicable diseases (NCDs) screening
  - Treatment for communicable diseases or public health threats e.g., tuberculosis or elephantiasis Treatment for NCDs e.g., diabetes and hypertension

Well-being e.g., work permits, visa extensions, housing purchases,....
